# Sphingolipids regulate neuromuscular synapse structure and function in *Drosophila*

**DOI:** 10.1002/cne.24466

**Published:** 2018-08-02

**Authors:** Ryan J. H. West, Laura Briggs, Maria Perona Fjeldstad, Richard R. Ribchester, Sean T. Sweeney

**Affiliations:** ^1^ Department of Biology and Hull York Medical School University of York Heslington York YO10 5DD UK; ^2^ Euan MacDonald Centre for Motor Neurone Disease Research and Centre for Discovery Brain Sciences University of Edinburgh Edinburgh EH8 9JZ UK

**Keywords:** lipid rafts, neuromuscular junction, synaptic adhesion, synaptic bouton, RRID:AB_2338959, RRID:AB_2713991, RRID:AB_2314867, RRID:AB_528203, RRID:AB_528484, RRID:AB_2166869, RRID:AB_330924, RRID:AB_10694715

## Abstract

Sphingolipids are found in abundance at synapses and have been implicated in regulation of synapse structure, function, and degeneration. Their precise role in these processes, however, remains obscure. Serine Palmitoyl‐transferase (SPT) is the first enzymatic step for synthesis of sphingolipids. Analysis of the *Drosophila* larval neuromuscular junction (NMJ) revealed mutations in the SPT enzyme subunit, *lace/SPTLC2* resulted in deficits in synaptic structure and function. Although NMJ length is normal in *lace* mutants, the number of boutons per NMJ is reduced to ∼50% of the wild type number. Synaptic boutons in *lace* mutants are much larger but show little perturbation to the general ultrastructure. Electrophysiological analysis of *lace* mutant synapses revealed strong synaptic transmission coupled with predominance of depression over facilitation. The structural and functional phenotypes of *lace* mirrored aspects of *Basigin* (*Bsg*), a small Ig‐domain adhesion molecule also known to regulate synaptic structure and function. Mutant combinations of *lace* and *Bsg* generated large synaptic boutons, while *lace* mutants showed abnormal accumulation of Bsg at synapses, suggesting that Bsg requires sphingolipid to regulate structure of the synapse. In support of this, we found Bsg to be enriched in lipid rafts. Our data points to a role for sphingolipids in the regulation and fine‐tuning of synaptic structure and function while sphingolipid regulation of synaptic structure may be mediated via the activity of Bsg.

**Abbreviations**
NMJ: Neuromuscular Junction
HRP: Horse Radish Peroxidase
TEM: Transmission Electron Microscopy
SPT1: Serine Palmitoyl Transferase
SPTLC1/2: Serine Palmitoyl Transferase Light Chain 1/2
EMS: ethyl methyl sulphonate
HSAN1: Hereditary Sensory and Autonomic Neuropathy Type 1
LSD: Lysosomal Storage Disease
TOF: Train of Five

## 1 INTRODUCTION

In 1967, Derry and Wolfe (1967) identified a prominent enrichment of glycosphingolipids within synaptic structures in the mammalian brain. To date our understanding of the role of these enigmatic lipids in synapse structure and function has yet to be fully elucidated. Sphingolipids are major lipid components of the plasma and endomembrane system and have been implicated in many forms of neuropathy and neurodegeneration (for review see Sabourdy et al., 2015). Sphingolipids are proposed to generate structure in membranes due to their rigidity and association with cholesterol (see Munro, 2003). They are also known to be potent signaling molecules regulating processes such as apoptosis, proliferation, migration, and responses to oxidative stress (reviewed in LLahiri & Futerman, 2007).

Numerous neurological and neurodegenerative conditions are directly attributable to the inability to synthesize or catabolize sphingolipids. The failure to synthesize all or particular sphingolipids gives rise to a number of neurological conditions such as infant‐onset symptomatic epilepsy (loss of GM3 ganglioside synthesis (Simpson et al., 2004), bovine spinal muscular atrophy (loss of 3‐ketohydrosphingosine reductase (Krebs et al., 2007) and hereditary sensory and autonomic neuropathy type 1, HSAN1, recessive and dominant mutations in serine palmitoyl transferase subunit 1 ((*SPTLC1*), Bejaoui et al., 2001; Dawkins, Hulme, Brahmbhatt, Auer‐Grumbach, & Nicholson, 2001). Conversely failure to catabolize sphingolipids in the lysosome generates a subset of lysosomal storage diseases/disorders (LSD's) known as sphingolipidoses, of which there are approximately 14 identified separate genetic conditions (reviewed in Kacher & Futerman, 2006). Sphingolipids are now suggested to have a prominent role in the onset and progression of Alzheimer's disease (Grimm et al., 2005) while the production after bacterial infection of autoimmune antibodies to gangliosides present at the neuromuscular synapse is likely to cause the dramatic and often lethal paralysis seen in Guillain‐Barré and Miller‐Fisher syndromes (Roberts, Willison, Vincent, & Newsom‐Davis, 1994; Willison et al., 1997). The presence of sphingolipids at the synapse is further attested by the ability of tetanus and botulinal toxins to effect their entry to synapses via co‐attachment to synaptic glycosphingolipids (Nishiki et al., 1996; Deinhardt, Berninghausen, Willison, Hopkins, & Schiavo, 2006).

While the presence of sphingolipids (in particular, glycosphingolipids) at the synapse is well established, little is known about their functional or structural role in the operational life of the synapse. Some in vitro studies have addressed the role of sphingolipids at synapses in the context of sphingolipid/cholesterol microdomains and indicate roles in the function and localization of neurotransmitter receptors, (Brusés, Chauvet, & Rutishauser, 2001; Hering, Lin, & Sheng, 2003) and synaptic exocytosis (Salaün, Gould, & Chamberlain, 2005; Darios et al., 2009; Chan & Sieburth, 2012; Chan, Hu, & Sieburth, 2012). The prominence of sphingolipids in neurological disease suggests that absence or accumulation of sphingolipids can exert an influence in synaptic function and indicates an inappropriately large gap in our knowledge regarding the actions of these lipids at the synapse. In the above outlined context, roles for sphingolipids in synapse structure and function remain to be determined. To this end, we have undertaken an analysis of sphingolipid function at a model synapse, the third instar neuromuscular junction of *Drosophila*. We have examined mutations in *SPT2*/*SPTLC2* (Serine Palmitoyltransferase, Long Chain Base Subunit 2), which encodes an essential subunit of the Serine Palmitoyltransferase (SPT) heterodimer necessary for the initial step in sphingolipid synthesis, for defects in neuromuscular synapse structure. We present evidence to suggest that sphingolipids are essential for synaptic structure and function, and structural regulation may be mediated partially through function of the Ig domain adhesion protein Basigin/CD147 (Bsg).

## 2 MATERIALS AND METHODS

### 2.1 Fly stocks and maintenance

*Drosophila* stocks were raised on standard yeast, sugar, and agar medium at 25°C (4% yeast, 8% sucrose, 1.2% agar, 3.6 mM calcium chloride, 0.65g/l ferrous sulphate, 6.5 g/l potassium sodium tartrate, 0.4 g/l sodium chloride and 0.4 g/l manganese chloride, 0.065% Nipagin, 0.0005% Bavistin). *Spt2/lace* alleles were a kind gift from John Roote (The University of Cambridge, UK), *hiw* stocks were gifted by Aaron DiAntonio (Washington University, USA) and *UAS‐lace‐HA*, *lace^k05305^* and *lace^2^* were a gift from Takashi Adachi‐Yamada (Gakushuin University, Japan), *Bsg* mutants and rescue transgenes were a gift from Anne Ephrussi (EMBL, Heidelberg). All other stocks were obtained from the Bloomington *Drosophila* Stock Center.

### 2.2 Immunohistochemistry

Third instar wandering larvae were dissected in PBS and fixed in 3.7% formaldehyde/PBS for 7 min. Following washes larvae were stained using the appropriate antibody in 0.1% PBT. Primary antibodies were used at the following concentrations Cy3 conjugated α‐HRP (1:200, goat, Jackson ImmunoResearch, Stratech Scientific), α‐syt (1:2,000, rabbit, West, Lu, Marie, Gao, & Sweeney, 2015), α‐nc82 (1:50, mouse, DSHB), α‐dlg (1:50, mouse, DSHB), α‐GluRIIb (1:2,500, rabbit, a kind gift from Aaron DiAntonio, Washington University, St Louis, MO (Marrus, Portman, Allen, Moffat, & DiAntonio, 2004; DiAntonio et al., 1999), α‐Bsg (1:200, Rat, a kind gift from Anne Ephrussi, EMBL, Heidelberg; Besse et al., 2007). Cy3, Cy5 and FITC conjugated secondary antibodies were used at 1:200 (goat, Jackson ImmunoResearch, Stratech Scientific). A comprehensive list of antibodies can be found in Supporting Information Table S1.

### 2.3 Electrophysiology

Intracellular microelectrode recordings were made from Muscles 6 and 7 in abdominal segments 3 and 4 of filleted third instar larval preparations bathed in HL3 saline, using standard techniques (Powers, Grizzaffi, Ribchester, & Lnenicka, 2016). The concentrations of Ca^2+^ and Mg^2+^ in the saline were reduced (to 0.4 and 10 mM, respectively) in order to depress mean quantal content of excitatory junction potential (EJP; evoked transmitter release), thereby increasing sensitivity to differences in synaptic strength between the *Drosophila* lines we tested (Dodge & Rahamimoff, 1967; Guan et al., 2017). The reduced EJP amplitude under these conditions also obviated correction of EJP amplitudes for non‐linear summation (McLachlan & Martin, 1981). Preparations were mounted in a recording chamber (bath volume approximately 1 ml) on the stage of an Olympus BX50WI upright, fixed stage microscope and visualized using 10× or 20× water‐dipping objectives. Glass capillary microelectrodes with resistances 15–40 MΩ were pulled using a Brown‐Flaming P87 puller (Sutter Instruments, Novato), filled with 3M KCl and mounted on an MP‐85 Huxley‐type micromanipulator (Sutter Instruments). The reference electrode was an Ag/AgCl pellet connected to the system ground. Membrane potentials were recorded using pClamp 10 (Clampex) software via an HS2A headstage (0.1× gain) connected to a Geneclamp 500 amplifier and Digidata 1550B interface (all Molecular Devices, Sunnyvale). Segmental nerves were aspirated into a micropipette with a heat‐polished tip, aperture 10–15 µm, and stimulated with trains of four or five (TOF) supramaximal pulses (nominally 10V, 0.1–0.2 ms duration; interval 50 ms, that is, 20 Hz; programmed in Clampex) triggering a DS2 stimulator (Digitimer, Welywn Garden City, UK). Three pulse trains were delivered at 5 s intervals and each train was preceded by either a positive or negative rectangular 100 ms, 1 nA current pulse delivered through the recording microelectrode. The voltage deflection (after subtraction of electrode resistance) was used to calculate input resistance and qualitatively check membrane time constant as indicators of membrane integrity. Recordings from muscles with input resistances less than 1.5 MΩ or time constant less than 5 ms were rejected (Powers et al., 2016). Spontaneous EJPs (miniEJPs) were recorded in the absence of nerve stimulation over a period of up to 60 s. EJP recordings were analyzed using pClamp 10.6 and miniEJPs were measured using Minianalysis (Synaptosoft, Atlanta). Mean frequency of miniEJPs was estimated from the inverse of their mean intervals. EJP and miniEJP amplitudes were corrected to an arbitrary standard membrane potential of −65 mV before calculating quantal content by the direct method (Ribchester, 2011). An index of synaptic facilitation (*f*: positive values indicating facilitation, negative values indicating depression) was calculated from the change in quantal content of the first (*m*_1_) and either the fifth (*m*_5_) or occasionally the fourth EJP, according to the formula *f = m*_5_/*m*_1_−1.

### 2.4 Imaging and quantification

Imaging and quantification of synaptic structure was performed as described in (West et al., 2015). Briefly, synaptic bouton numbers at muscles 6/7 hemisegment A3, were determined by counting each distinct, spherical, anti‐synaptotagmin‐positive varicosity contacting the muscle. As synaptic bouton number has been shown to increase proportionally with muscle surface area synaptic bouton numbers were normalized against muscle surface area by dividing the bouton number by the muscle surface area and multiplying by mean wild‐type muscle surface area as described by (Milton et al., 2011). Muscles and synapses were imaged at room temperature using a camera (AxioCam HRC) on an inverted fluorescence microscope (Axiovert 200; Carl Zeiss) using Plan Neofluar 10×/0.3 NA and 40×/0.75 NA lenses, with Axio‐ Vision Rel. 4.8 software (Carl Zeiss). Measurements were made from images using ImageJ (National Institutes of Health). Confocal images were obtained using a confocal microscope (LSM 710 Axio Observer Z1; Carl Zeiss). Z‐stacked images of single NMJ's were obtained using a Plan Apochromat 63×/1.4 NA oil objective. Z‐stack projections of muscle 4 NMJ's were analyzed using ImageJ to quantify bouton diameter, NMJ length, and satellite bouton number. Bouton diameter was measured as the width across a bouton at the widest point (Milton et al., 2011). NMJ length was measured using the NeuronJ ImageJ plugin.

Transmission electron microscopy was performed as described previously in (West et al., 2015). Third instar wandering larvae were dissected and fixed in 0.1 M NaPO4, pH 7.4, 1% glutaraldehyde, and 4% formaldehyde, pH 7.3, overnight. Fixed larval preparations were washed 3× in 0.1 M NaPO_4_ before incubation in OsO_4_ (1% in 0.1 M NaPO_4_; 2 hr). Preparations were washed 3× in distilled water before incubation in 1% uranyl acetate. Preparations were washed (3× distilled water) and dehydrated through a graded ethanol series; 20% increments starting at 30% followed by two 100% changes and then 2× 100% propylene oxide. Preparations were incubated in a graded series of epon araldite resin (in propylene oxide); 25% increments culminating in 3× 100% changes. Individual muscles were then dissected out. These were then transferred into embedding molds, and the resin was polymerized at 60°C for 48 hr. Resin mounted preparations were sectioned (60–70 nm) using glass knives upon a microtome (Ultracut UCT; Leica) and placed onto grids. Preparations were subsequently incubated in uranyl acetate (50% in ethanol), washed in distilled water, and incubated in lead citrate. Sections were imaged using a transmission electron microscope (TECNAI 12 G^2^; FEI) with a camera (Soft Imaging Solutions MegaView; Olympus) and Tecnai user interface v2.1.8 and analySIS v3.2 (Soft Imaging Systems). Quantification of active zone length, number of synaptic vesicles localized within 250 nm of the T‐bar active zone, synaptic vesicle diameter and mitochondrial size was performed using ImageJ. Representative images were taken from at least three animals per genotype.

### 2.5 Lipid raft extraction and Western blotting

Methodology for purification of lipid rafts was adapted from (Fernandez‐Funez et al., 2009) and (Zhai, Chaturvedi, & Cumberledge, 2004). Briefly 50 third instar larvae (w^1118^) were sonicated in 250 μl cold TNET buffer (100 mM Tris, 0.2 mM EGTA, 150 mM NaCl, 0.3 M Sucrose, pH 7.5, 1% Triton‐X, 1x protease inhibitor) and incubated on ice for 30 min. Debris was removed by centrifugation at 3000*g* for 10 min. and 200 μl of crude supernatant extract mixed with 400 μl of 60% Optiprep^TM^ in 5 ml. 5% Optiprep^TM^ was underlaid with 1.8 ml 30% Optiprep^TM^ which was underlaid by the Optiprep^TM^ and extract mixture in 5.1 ml ultracentrifuge tubes. Gradients were spun at 43,865 RPM for 1 hr at 4°C in a Beckman Coulter Optima^TM^ L‐100 XP Ultracentrifuge using a VTi90 Rotor. Following centrifugation 10 500 μl fractions were collected from the bottom and analyzed via western blotting. Antibodies against the α Subunit of the Na^+^/K^+^ ATPase (1 : 100,000, mouse, DSHB) and Syntaxin (1:50, mouse, DSHB) were used as negative and positive controls for lipid rafts, respectively. Anti‐Bsg (1:1,500, rat) was a kind gift from Dr. Anne Ephrussi (EMBL Heidelberg, Germany). HRP‐conjugated secondary antibodies were from Cell signaling technology. A comprehensive list of antibodies can be found in Supporting Information Table S1.

## 3 RESULTS

### 3.1 SPTLC2/lace function is essential in Drosophila

**Figure 1:**
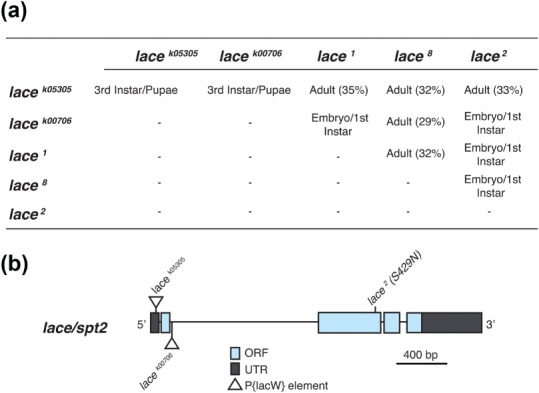
lace is essential for survival in *Drosophila*. (a) Complementation analysis between *lace* mutants was used to identify allelic combinations that gave an optimal penetration, generating an early/late pupal lethality, for analysis of the 3rd instar neuromuscular junction. Percentages shown represent the number of unbalanced transheteroygous flies that eclosed for those crosses where transheterozygotes survived to adulthood, baseline = 50%. (b) Gene schematic showing the location of the two *P*‐element insertions mapped to the lace locus in this study (*l(2)lacW^K05305^* and *l(2)lacW^K00706^*) as well as the previously mapped EMS induced *lace^2^* mutation

SPT acts in the first enzymatic step in the de novo synthesis of sphingolipids, catalyzing the condensation of l‐serine with palmitoyl‐coA to generate 3‐ketosphinganine and further subsequent sphingolipid derivatives. SPT is composed of two subunits, SPTLC1 and SPTLC2. Previously we have demonstrated that expression of *Drosophila* SPTLC1 (dSPT1) bearing a neomorphic mutation associated with HSAN1, and aberrant sphingolipid production, induced morphological aberrations in synapse growth at the *Drosophila* third instar larval NMJ (Oswald, West, Lloyd‐Evans, & Sweeney, 2015). In order to determine the role of sphingolipids in the regulation of synaptic morphology we looked to characterize further the role of *SPTLC2* in regulating the structure and growth of the *Drosophila* larval neuromuscular synapse. Lethal and hypomorphic mutations in the *Drosophila SPTLC2* gene, *lace*, have previously been identified (Ashburner, 1982; Adachi‐Yamada et al., 1999). Here, using iPCR, we mapped the insertion sites of the two *P*‐element insertions within the *lace* locus (Figure 1). *l(2)lacW^k05305^* was mapped to 97 nucleotides upstream of the start ATG codon while *l(2)lacW^K00706^* mapped 52 nucleotides downstream of the first exon (Figure 1b). The *lace^2^* allele has previously been identified as an EMS induced point mutation leading to the amino acid change S429N (Sasamura, Matsuno, & Fortini, 2013). The *lace^8^* allele is an additional EMS induced mutation while *lace^1^* is a spontaneous mutation (Ashburner, 1982). Using this series of mutants, we screened for allelic combinations that generated an early/late pupal lethality (Figure 1a), giving an optimal penetration of the phenotype and a reduction in sphingolipid content for an analysis of the 3rd instar NMJ. *Lace^k05305^/lace^2^* transheterozygotes and *lace^k05305^* homozygotes were identified as giving an optimal lethal phase for studying synaptic growth and structure. In addition it has previously been demonstrated that l*ace^k05305^/lace^2^* transheterozygotes and *lace ^k05305^* homozygotes present with just 5.5% and 2.5% sphingolipid content (Herr et al., 2003), respectively, compared to wildtype. A schematic of the *lace* alleles utilized in this study is given in Figure 1b. All mutant combinations were found to be lethal.

### 3.2 Lace mutants display aberrant NMJ structure

**Figure 2:**
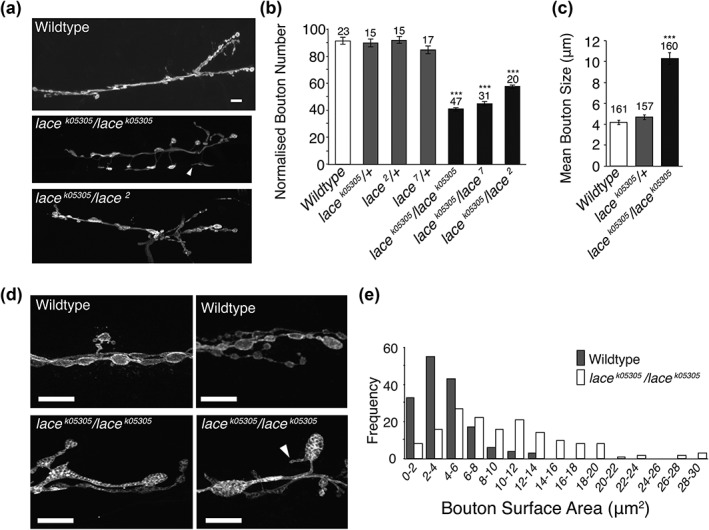
Loss of sphingolipid synthesis leads to enlarged bouton structure at the NMJ. (a, b) *Drosophila* third instar larvae presenting with homozygous or transheterozgous mutations in *lace* display a significant reduction in synaptic bouton number (ANOVA *p* < .001, with post‐hoc Dunnett's comparison to wildtype controls: *** *p* < .001). (c–e) Reduced synaptic bouton number is coupled with a significant increase in mean synaptic bouton size, associated with an increased frequency of synaptic boutons displaying a surface area > 8 μm^2^ in lace mutants. *lace* mutants also displayed spur like protrusions from terminal boutons (arrow heads, a and d). Scale bars = 10 μm

**Figure 3:**
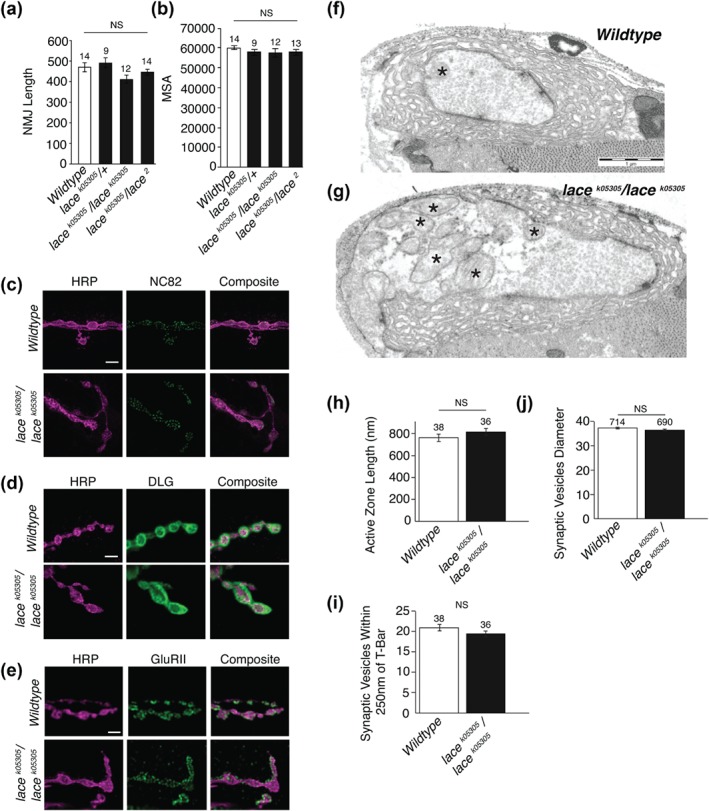
Synaptic components appear unchanged in *lace* mutants. (a, b) *lace* mutants show no change in total NMJ length or muscle surface area. a; ANOVA; *p* < .064, b; ANOVA; p < .616. (c–e) Pre‐ (nc82) and post‐ (discs large and GluRII) synaptic markers appear unchanged at an immunohistochemical level in lace mutants. Scale bars = 5 μm. (f–j) Ultrastructural examination reveals no significant perturbation to active zone length (h), synaptic vesicle number (i) or synaptic vesicle diameter (j). Enlarged mitochondria (asterisk) are observed in lace mutants (g). Scale bars = 1 μm

Using the *Drosophila* third instar larval NMJ as a model synapse we identified both transheterozygous and homozygous *lace* mutants displaying significant perturbations to gross morphological NMJ structure. This was characterized by a significant decrease in synaptic bouton number coupled with an increase in bouton size (Figure 2a–e). It was also observed that mutants showed the presence of spur‐like structures emanating from terminal boutons (Figures 2d), suggesting either partially formed synaptic extensions, or collapse of a bouton. *Lace* mutants showed no significant variance in the length of the NMJ arbour or muscle surface area when compared to wildtype (Figure 3a, b).

Despite significant perturbations at the gross morphological level, at a sub‐cellular level pre‐ and post‐synaptic structures appear unperturbed, with no observable alteration to either the pre‐synaptic active zone marker nc82/bruchpilot or post‐synaptic markers GluRIIA and Discs‐large (DLG) (Figure 3c–e). There was also no difference in Futsch or FasII (Supporting Information Figure S1). Mutants did, however, show an apparent disruption to plasma membrane antigens recognized by the anti‐horse radish peroxidase (HRP) antibody, leading to an uneven distribution of HRP labelling (Figures 2d, 3c–e, & Supporting Information Figure S2a). Perturbed HRP staining was not observed in wildtype animals.

At ultrastructural level individual synaptic components also appear normal (Figure 3f–j), showing no significant aberration to active zone size, synaptic vesicle number or synaptic vesicle size (Figure 3h–j). One notable observation, however, is the number of enlarged mitochondria observed throughout the nervous system of *lace* mutants (Figure 3g, Supporting Information Figure S2). No significant difference in the total number of mitochondria was observed between genotypes (data not shown). Taken together these initial findings suggest that sphingolipid is essential for maintaining synapse structure at a gross morphological level.

### 3.3 NMJ length is maintained with fewer boutons in the absence of sphingolipids

**Figure 4:**
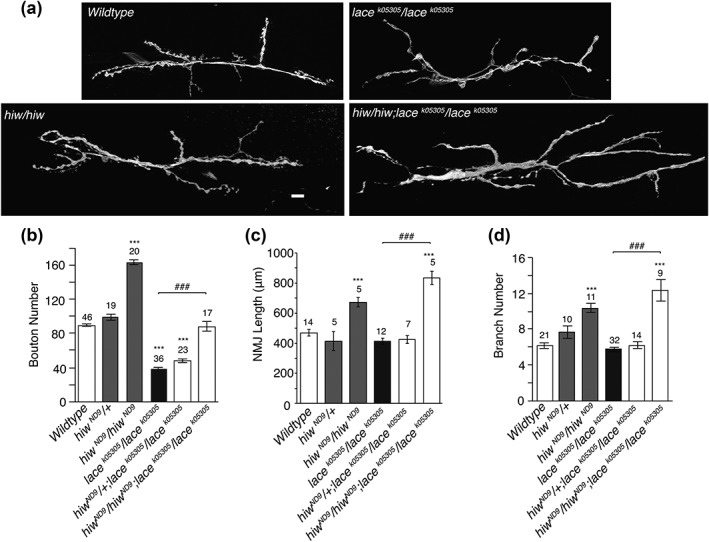
*lace* mutants are capable of further synaptic growth. (a, b) Combining *lace* mutants with the synaptic overgrowth mutant *highwire* revealed double mutants show a ∼50% reduction in bouton number, compared to *Hiw* alone. This is comparable to the ∼50% reduction observed in *lace* mutants, compared to wild type. *Hiw;lace* double mutants, however, remain capable of further synaptic growth showing a significant increase in synaptic length (c) and branching (d), comparable to that seen in *Hiw* single mutants. ANOVA *p* < .001, with post‐hoc Dunnett's comparison to wildtype controls: *** *p* < .001 and Tukey between group's comparison: ### *p* < .001

Boutons are added to the neuromuscular junction during progression through the larval instars (Zito, Parnas, Fetter, Isacoff, & Goodman, 1999). The counting of boutons commonly stands proxy for NMJ size in many studies of the larval neuromuscular junction (Schuster, Davis, Fetter, & Goodman, 1996). On initial observation, the *lace* mutant NMJ appeared to be of normal length despite having a reduced bouton count. Having identified a defect in bouton structure, we then examined NMJ length in relation to bouton structure. By counting boutons per NMJ arbour while simultaneously measuring the length of the NMJ we found that in sphingolipid depleted NMJ's the overall length of the NMJ was indistinguishable from wildtype while boutons per NMJ was found to be significantly reduced by ∼50%, compared to wild‐type (Figures 2 and 4a–c). *lace* mutants showed no reduction in muscle surface area (Figure 3b), indicating that reduced bouton number was not as a result of reduced muscle size. Branching patterns of NMJ's were also indistinguishable between sphingolipid depleted NMJ's and wild‐type (Figure 4d).

To ascertain whether sphingolipid deficient *lace* mutant NMJ's were capable of further synaptic growth, we combined the *lace^k05305^/lace^k05305^* mutant with the synaptic overgrowth mutant *highwire* (*hiw*) (Wan et al., 2000; Collins, Wairkar, Johnson, & DiAntonio, 2006). NMJ's in the *hiw^ND9^;lace^k05305^/lace^k05305^* mutant combination were found to be capable of growth well above wildtype length (Figure 4). In the *hiw/lace* mutant combination, boutons per unit‐length were generated at around 50% of the *hiw* figure alone. This is similar to the comparison between wild‐type and *lace* where *lace* produces ∼50% less boutons per unit length compared to wild‐type. Collectively this data indicates a defect in NMJ synaptic structure, but not overall NMJ length regulation, in the absence of sufficient sphingolipid.

**Figure 5:**
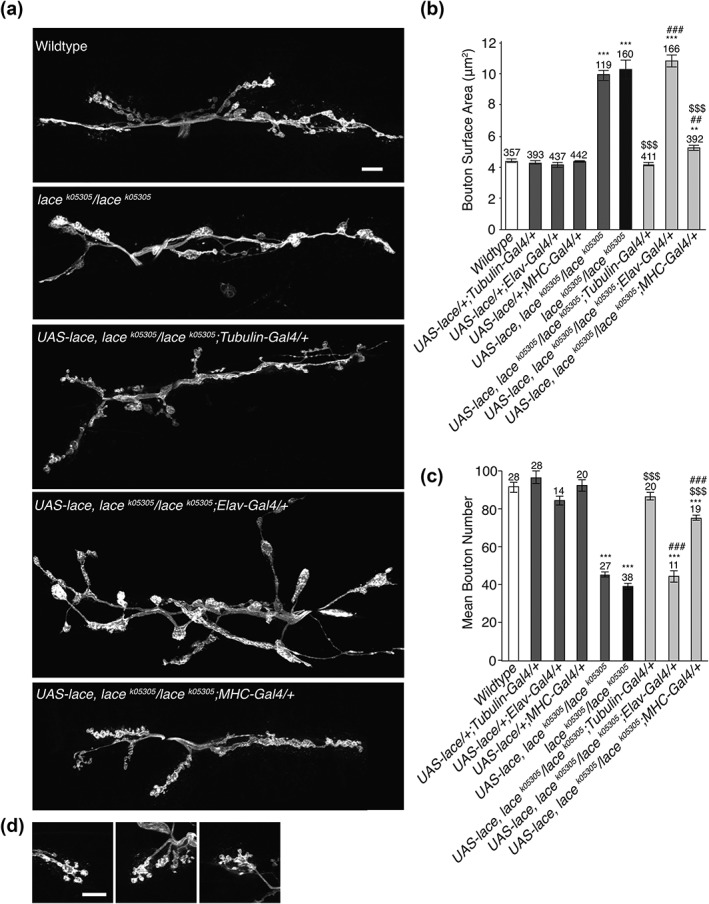
Aberrant synaptic architecture can be partially alleviated by post synaptic expression of lace. (a–c) Global (Tubulin‐Gal4) expression of UAS‐*lace* was sufficient to completely alleviate both the increase in synaptic bouton size (b) and reduction in synaptic bouton number (c) observed in *lace* mutants. Post‐synaptic (*MHC*‐Gal4) expression of UAS‐*lace* elicits an almost complete rescue of both enlarged synaptic bouton size and reduced bouton number. Pre‐synaptic (*Elav*‐Gal4) expression of lace is insufficient to rescue aberrant synaptic architecture. (a) ANOVA; *p* < .001 with post hoc Dunnett's comparison to wildtype: *** *p* <.001, ** *p* < .01 and Tukey comparison within groups vs Gal4 control ### *p* <.001, ## *p* < .01 or vs lace mutant $$$ *p* <.001. (b) ANOVA; *p* < .000 with post hoc Dunnett's comparison to wildtype: *** *p* <.001, and Tukey comparison within groups vs Gal4 control ### *p* <.001 or vs lace mutant $$$ *p* <.001

### 3.4 Expression of lace rescues synaptic structure

Having ascertained that sphingolipid is essential to the generation or maintenance of mature synaptic structure, we examined the relative sphingolipid contribution of the pre‐ and post‐synaptic compartments. To facilitate this, we employed the *lace^k05305^/lace^k05305^* mutant and rescued lace function in either the pre‐synaptic compartment alone, using the pan‐neuronal *elav*‐GAL4 driver, or the post‐synaptic compartment alone, using the muscle expressing *MHC*‐GAL4 driver. We also performed a global rescue using *Tubulin*‐GAL4 driven expression of UAS‐*lace*. Here we found that presynaptic expression of lace (*elav*‐GAL4) failed to recover bouton structure or number in the *lace^k05305^/lace^k05305^* background (Figure 5). In contrast rescue of lace function in the post‐synaptic muscle compartment induced a nearly complete rescue of both reduced synaptic bouton number and bouton enlargement (Figure 5). Global expression of UAS‐*lace* was sufficient to completely rescue all aspects of NMJ perturbation in *lace* mutants (Figure 5). We also examined a role for glia in the sphingolipid regulation of NMJ structure. Glial expression of lace, using the *repo*‐gal4 driver, was sufficient to rescue both reduced synaptic bouton number and enlarged bouton size in *lace^k05305^/lace^k05305^* mutants (Supporting Information Figure S3). Interestingly the post‐synaptic rescue of lace function induced the formation of excessive “satellite” boutons (Figure 5d). Satellite boutons are small boutons sprouting from the main synaptic arbour (Beumer, Rohrbough, Prokop, & Broadie, 1999; Koh, Verstreken, & Bellen, 2004; Marie et al., 2004). These data suggest a partial non‐cell‐autonomous role for sphingolipids in the regulation of synaptic growth and structure. Feeding *Drosophila* larvae sphingosine, the product of serine palmitoyl transferase activity can rescue some phenotypes caused by loss of SPT (Adachi‐Yamada et al., 1999). Our data points to an ability to rescue the sphingolipid deficiency NMJ phenotype with global, muscular or glial expression, but not neuronal expression.

### 3.5 Lace mutants show increased synaptic strength

Sphingolipids have previously been implicated in the synaptic vesicle cycle (Salaün et al., 2005; Darios et al., 2009; Chan & Sieburth, 2012; Chan et al., 2012) and in the localization and function of neurotransmitter receptors (Brusés et al., 2001; Hering et al., 2003). Having observed significant perturbations to NMJ morphology, but not ultrastructure, we carried out an electrophysiological analysis in *lace* mutants to determine the role that sphingolipids might play in the regulation of synaptic activity.

**Figure 6:**
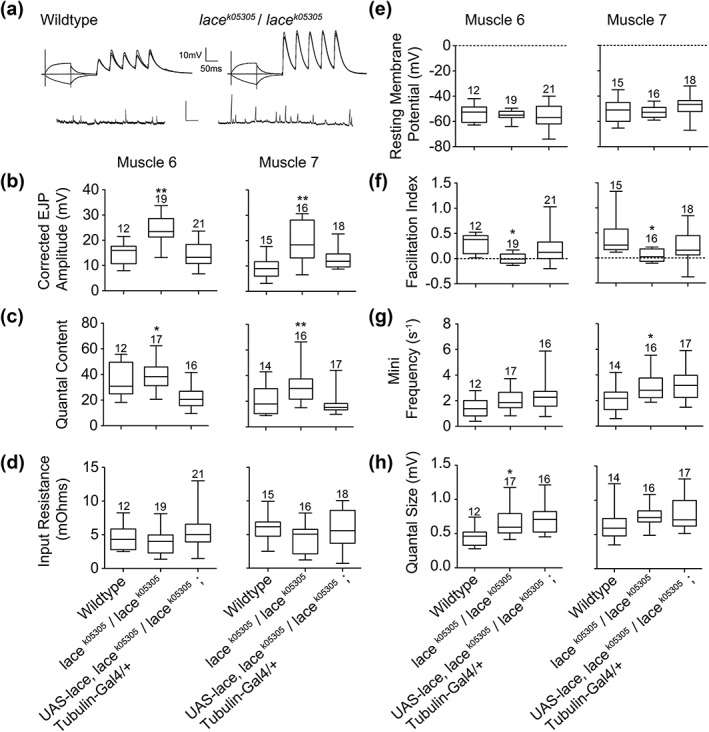
*lace* mutants display increased synaptic strength. (a) representative intracellular recording traces, showing evoked EJP responses to TOF stimulation and spontaneous (mini) EJPs. (b) *lace* mutant (*lace^k05305^*/*lace^k05305^*) larvae show significantly (ANOVA with post‐hoc Tukey comparison between groups, ** *p*<.01) elevated evoked EJP amplitudes at muscles 6 and 7 compared to wildtype larvae and larvae expressing *lace* globally (tubulin‐gal4) in a *lace* mutant background (rescues). Box and whisker plots demonstrate the median, interquartile range and the range of recorded values. (c) *lace* mutant (*lace^k05305^*/*lace^k05305^*) larvae show a significant increase (ANOVA with post‐hoc Tukey comparison between groups, * *p*<.05, ** *p*<.01) in quantal content at muscles 6 and 7 compared to wildtype larvae and larvae expressing *lace* globally (tubulin‐gal4) in a *lace* mutant background (rescues). Box and whisker plots demonstrate the median, interquartile range and the range of recorded values. (d, e) No significant variance in input resistance or resting membrane potential was observed between genotypes. (f) Synaptic facilitation index (*f*: positive values indicating facilitation, negative values indicating depression) calculated from the change in quantal content of the first (*m*_1_) and either the fifth (*m*_5_) or occasionally the fourth EJP, according to the formula *f*=*m*_5_/*m*_1_−1. Box and whisker plots demonstrate the median, interquartile range and the range of recorded values (ANOVA with post‐hoc Tukey comparison between groups, * *p*<.05). (g) *lace* mutant (*lace^k05305^*/*lace^k05305^*) larvae show a significant (ANOVA with post‐hoc Tukey comparison between groups, * *p*<.05) increase in spontaneous (mini) release frequency at muscle 7 compared to wildtype larvae. Box and whisker plots demonstrate the median, interquartile range and the range of recorded values. (h). *lace* mutant (*lace^k05305^*/*lace^k05305^*) larvae show a significant (ANOVA with post‐hoc Tukey comparison between groups, * *p*<.05) increase in quantal size at muscle 6, compared to wildtype larvae. Box and whisker plots demonstrate the median, interquartile range and the range of recorded values

Mutant *lace* larvae showed a significant increase (∼50%) in both evoked EJP amplitude (Figure 6a, b, Supporting Information Tables S2 and S3) and quantal content (Figure 6c) at both muscles 6 and 7, compared to wild‐type controls. No significant difference in input resistance or resting membrane potential was observed between genotypes (Figure 6d, e). Expression of lace under the control of the global driver *tubulin*‐Gal4 was sufficient to alleviate both elevated evoked EJP amplitude and quantal content in *lace* mutant larvae (Figure 6b, c). At the Ca^2+^/Mg^2+^ concentrations used in the present experiments, consistent synaptic facilitation during Train‐of‐Five (TOF) stimulation was observed in wildtype larvae but not in *lace* mutants (Figure 6f). Specifically, *lace* mutant larvae show a consistent and constant EJP size during the TOF stimulus at NMJs in both muscles 6 and 7, compared to wild‐type larvae (Figure 6f). This difference in EJP consistency during the TOF stimulus was partially rescued by global (*tubulin*‐Gal4) expression of lace in the *lace* mutant background (Figure 6f). Mutant *lace* synapses also showed a significant increase in mini frequency (Muscle 7, Figure 6g) and Quantal size (Muscle 6, Figure 6h), compared to wildtype. These phenotypes, however, were not rescued by expression of wildtype lace (*tubulin*‐Gal4, Figure 6g, h).

### 3.6 Lace mutants reveal a relationship between lace and Basigin in the regulation of synapse structure

Previous studies have identified that the Ig family protein Basigin/CD147 (Bsg) is required pre‐ and post‐synaptically to restrict synaptic bouton size and regulate bouton number. *Bsg* mutants were shown to display significantly enlarged boutons and a reduction in synaptic bouton number, while the overall NMJ size remained close to wild‐type (Besse et al., 2007), a phenotype similar to *lace*. *Bsg* mutants also show an elevated evoked EJP amplitude, mini amplitude and mini frequency similar to *lace* mutants (Besse et al., 2007) with an additional release asynchrony. As with sphingolipids, *Bsg* has also been implicated in the regulation of actin cytoskeleton dynamics, with mutants showing accumulation of mitochondria (Curtin, Meinertzhagen, & Wyman, 2005), perturbed synaptic structure and an early lethal phase (Besse et al., 2007). As such we asked whether a functional interaction existed between *Bsg* and the loss of sphingolipid function generated in *lace* mutants and whether Bsg localization was also altered in *lace* mutants.

**Figure 7:**
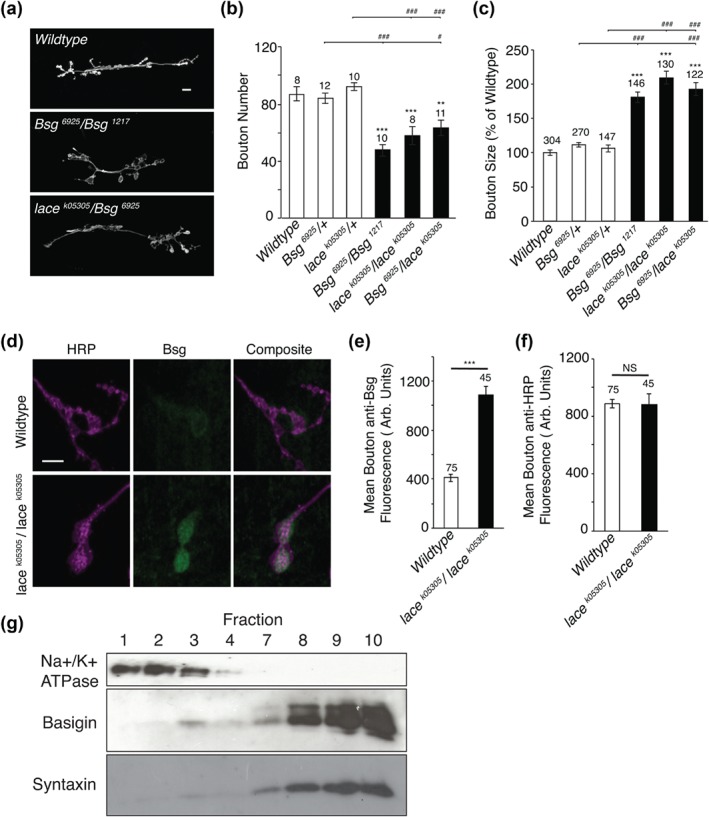
*lace* mutants reveal a functional interaction between lace and Basigin. (a–c) Heterozygous *lace/Bsg* double mutants phenocopy both *lace* and *Bsg* transheterozygotes, displaying a significant reduction in synaptic bouton number and enlargement of synaptic boutons. ANOVA; *p* < .001 with post hoc Dunnett's comparison to wildtype: *** *p* < .001, ** *p* < .01 and Tukey comparison within groups ### *p*< .001, # *p* < .05. (d–f) Bsg was shown to accumulate at NMJs in *lace* mutants, showing a significant increase in mean anti‐Bsg fluorescence compared to wildtype. Mean relative HRP fluorescence was also quantified, as a control, and showed no variance from wildtype. Student's *t*‐test; *p* < .000 (**d**) and *p* < .745 (e). (g) Gradient fractionation revealed to Bsg to be present within floating fractions 9 and 10, along with the lipid raft marker Syntaxin, demonstrating Bsg to be present in lipid raft microdomains in *Drosophila* larvae

Here we show that heterozygous *lace/Bsg* mutant combinations phenocopy both *lace* and *Bsg* mutants, displaying an ∼50% reduction in synaptic bouton number, coupled with significantly enlarged synaptic boutons (Figure 7a–c). As has previously been shown (Besse et al., 2007) heterozygous mutations in either *Bsg* or *lace* alone show no variance from wildtype.

Having identified an apparent genetic relationship between *Bsg* and *lace* we next looked to determine the abundance and localization of Bsg in *lace* mutants. Here we show that, as previously identified, Bsg is present at the NMJ. However, we also demonstrate there to be a significant increase in the amount of Bsg accumulating at *lace* mutant NMJ's when compared to wildtype (Figure 7d–f). Relative HRP was also quantified as a control (Figure 7f) with no significant variance observed between *lace* mutants and wildtype.

Sphingolipids are major constituents of lipid rafts, specialized membrane microdomains that act to regulate membrane dynamics, endocytic process and cell signaling events, amongst other processes. Previous studies have implicated Bsg in the regulation of signaling complexes within lipid‐raft like domains in cancer (Grass, Tolliver, Bratoeva, & Toole, 2013). We then proposed that the accumulation of Bsg observed at the NMJ of *lace* mutants may relate to its presence within lipid raft microdomains. To determine whether Bsg was present in microdomains in *Drosophila*, lipid rafts were isolated via optiprep gradient fractionation. The presence of Bsg within lipid raft fractions was confirmed by identification of its presence within floating fractions 9 and 10, which were also positive for the known lipid raft marker Syntaxin (Figure 7g; Chamberlain, Burgoyne, & Gould, 2001; Lang et al., 2001). The transmembrane ion pump Na+/K+ ATPase, which is excluded from lipid rafts and enriched in non‐lipid raft membranes (Fernandez‐Funez et al., 2009), was not. Taken together these findings suggest that Bsg is localized within lipid raft micro‐domains and that a functional interaction exists between sphingolipids and Bsg in the regulation of synaptic structure.

## 4 DISCUSSION

### 4.1 The role of sphingolipids at synapses

The enrichment of sphingolipids at synapses has been long known (Derry & Wolfe, 1967). Assigning functions for these enigmatic lipids at the synapse has remained problematic. Ablation of gangliosides in mouse has identified subtle defects in neurotransmission (Zitman et al., 2010, 2008, 2011) while loss of G3‐ganglioside synthesis results in an infantile onset epilepsy (Simpson et al., 2004), the mechanism for which remains obscure. A specific role for sphingosine has been identified in promoting SNARE protein fusion and synaptic exocytosis (Darios et al., 2009).

Many sphingolipid species present in the outer leaflet of the plasma membrane are found in association with cholesterol as “lipid rafts.” Neurons receive supplementary cholesterol from glia which is essential for supporting synapse maturation and additional synaptogenesis (Mauch et al., 2001) suggesting cholesterol, and potentially lipid rafts, are rate limiting for these processes. Depletion of both cholesterol and sphingolipids together has been shown to reduce and enlarge dendritic spines with eventual loss of synapses in hippocampal neurons in culture possibly due to reduced association with lipid rafts of synapse structure promoting proteins such as Post‐Synaptic Density protein 95 (PSD95) (Hering et al., 2003). In this present study, we have reduced synthesis of sphingolipids with a mutation in *SPTLC2* and examined the development of neuromuscular synapses in the *Drosophila* larval preparation. This approach has allowed us to study the genetic depletion of sphingolipids at an identified synapse in vivo and investigate a role for sphingolipids in the regulation of synaptic structure and activity. As part of this study, we have also identified a potential role for the Ig domain cell adhesion protein Bsg in sphingolipid dependent regulation of synaptic structure.

### 4.2 Sphingolipids are required for normal synapse structure

On examination of sphingolipid deficient synapses, we observed a disruption to the normal synaptic structure. We found that synaptic boutons were enlarged and the overall numbers of boutons reduced by ∼50% while the length of the neuromuscular synapse remained indistinguishable from wildtype. This phenotype is highly reminiscent of the reduction of synapse number, but increase in synapse size observed in hippocampal neurons in culture depleted for lipid rafts (Hering et al., 2003). Nevertheless, we were surprised that beyond the structural deficit of the synapse, the ultrastructure of the synapse was remarkably intact, suggesting a role in fine‐tuning of synaptic properties.

Synapses depleted for sphingolipids were capable of greater growth when combined with the synaptic overgrowth mutation *highwire* (*hiw*) (Wan et al., 2000). Our data suggests the mutations in *lace* and sphingolipid depletion decouples bouton structure from normal synaptic length. Large boutons are observed in mutants of *mothers against dpp (mad), thick veins (tkv), saxophone (sax) medea (med)*, and *glass‐bottom‐boat (gbb)*, components of the TGF‐ß pathway that is known to regulate synaptic growth (Aberle et al., 2002; Sweeney & Davis, 2002; Rawson, Lee, Kennedy, & Selleck, 2003; McCabe et al., 2004). However these mutations reduce synaptic length by ∼50% and ultrastructural synaptic defects such as nonplasma membrane attached active zones (T‐bars), large endosomal vesicles and ripples in pre‐synaptic peri‐active membranes are observed (McCabe et al., 2004). One obvious ultrastructural defect that is present in sphingolipid depleted synapses is enlarged mitochondria (Figure 3g, Supporting Information Figure S2). Enlarged mitochondria are observed in a number of sensory neuropathies (see Vital & Vital, 2012; for review) and it is of interest that dominant mutations in *SPTLC1* (Bejaoui et al., 2001; Dawkins et al., 2001) and SPTLC2 (Penno et al., 2010; Oswald et al., 2015) that generate aberrant sphingolipids give rise to Hereditary and Sensory Neuropathy Type 1 (HSAN1) where enlarged mitochondria are often observed. This may be attributable to a recognized role for sphingolipids in mitochondrial fission (Ciarlo et al., 2010).

To dissect the spatial requirement for sphingolipid regulation of synapse structure we rescued the *lace* mutant with a rescue transgene, expressed globally, pre‐ or post‐synaptically. We found that we could rescue synaptic bouton size and number with a global expression of the rescue transgene (Figure 5), but no aspects of the phenotype could be rescued with a pre‐synaptic expression. Perturbed NMJ morphology could also be rescued by glial or post‐synaptic expression of *lace*, however post‐synaptic muscle expression generated a partial rescue, with an excess of “satellite” boutons, a phenotype normally associated with integrin dysfunction (Beumer et al., 1999) or endocytic defects (Koh et al., 2004; Marie et al., 2004). Previous data feeding *lace* mutant larvae with sphingosine, the product of the SPT enzyme, partially rescued *lace* mutant associated phenotypes (Adachi‐Yamada et al., 1999). Taken together with our analysis, there is a strong suggestion that sphingolipid precursors such as sphingosine may be able to act non‐cell autonomously, and traffic between cells to support synapse structure and function, but not when supplied from the nervous system.

### 4.3 Sphingolipids are required for regulation of synaptic output

Analysis of EJP and miniEJP characteristics at the 3rd instar larval NMJ reveals mutations in *lace* produce, at the Ca^2+^/Mg^2+^ concentrations we used, a small but significant increase in synaptic strength, accompanied by a change in short‐term plasticity, with synaptic depression predominating over synaptic facilitation. NMJs with high‐quantal content EJPs normally show synaptic depression during paired or short‐train repetitive stimulation, while those with a low basal quantal content show synaptic facilitation (Lnenicka & Keshishian, 2000; Lnenicka, Theriault, & Monroe, 2006). Further analysis is required, for instance using a range of Ca^2+^ concentrations, to establish whether this apparent change in synaptic plasticity is commensurate with a greater basal synaptic strength in the *lace* mutant larvae, or whether it represents a specific effect of the mutation, disrupting the normal link between mechanisms that couple basal quantal content to short‐term synaptic plasticity.

In vitro and in vivo analysis has suggested a role for sphingolipids in synaptic vesicle endocytosis (Salaün et al., 2005; Shen et al., 2014) and exocytosis (Darios et al., 2009; Chan & Sieburth, 2012; Chan et al., 2012) in addition to a role in neurotransmitter distribution (Brusés et al., 2001; Hering et al., 2003). We observe no evident defects in neurotransmitter receptor distribution. Interestingly, ablation of major subsets of gangliosides and subsequent analysis of synaptic function at the NMJ in a mouse model reveals a more pronounced run‐down of neurotransmitter release upon sustained stimulation, consistent with the data we have presented here (Zitman et al., 2008, Zitman et al., 2011). We cannot however, directly attribute the apparent deficit in synaptic facilitation we observed here in *lace* mutants to exo‐ or endocytosis, at this point.

### 4.4 Sphingolipids interact with Basigin to regulate synaptic structure at the synapse

We noted a strong phenotypic similarity at the larval neuromuscular synapse between *lace* mutants and mutations in the small Ig domain adhesion protein *Basigin/CD147* (Besse et al., 2007). Bsg is a glycoprotein localized in the plasma membrane that is known to genetically interact with integrins (Curtin et al., 2005) during development of the *Drosophila* eye. In *Bsg* mutants, synaptic boutons at the larval neuromuscular junction are enlarged in size and reduced in number with a modest reduction in synaptic span (Besse et al., 2007). Bsg has previously been localized to sphingolipid enriched lipid rafts in invading epithelial breast cells (Grass et al., 2013) and we observed that Bsg is abundant in the lipid raft associated membrane fraction, co‐sedimenting with syntaxin, a known component of lipid rafts (Fernandez‐Funez et al., 2009). We cannot say at this juncture if Bsg function is directly regulated by sphingolipids. Indeed, recruitment of Bsg to lipid rafts can be critical for the recruitment of other protein factors such as claudin‐5 in retinal vascular epithelial cells (Arima et al., 2016). However, given the genetic interaction between *Bsg* and *lace*, with *bsg;lace* transheterozygous double mutants phenocopying both *lace* and *bsg* mutants, our data suggests Bsg and sphingolipids genetically interact to regulate synaptic structure. We interpret this interaction as indirect; the loss of sphingolipid generated in the *lace* mutant affecting Bsg function to regulate synapse structure and function

Synaptic sphingolipids have previously been implicated in synaptic vesicle release (Darios et al., 2009; Chan & Sieburth, 2012; Chan et al., 2012), endocytosis (Salaün et al., 2005), neurotransmitter receptor localization (Hering et al., 2003; Brusés et al., 2001) and maintenance of synaptic activity (Zitman et al., 2008, Zitman et al., 2011). However other roles at the synapse for these enigmatic lipids remain elusive. Two potential functions for sphingolipid at the synapse are suggested by our study. Mitochondrial uptake of Ca^2+^ shapes Ca^2+^ dependent responses (Mammucari et al., 2018). The enlarged mitochondria we observe in *lace* mutants may impinge on Ca^2+^ uptake to affect synaptic facilitation. A further deficit in Ca^2+^ handling at the synapse is suggested by the recent finding that Bsg is an obligatory subunit of plasma membrane Ca^2+^‐ATPases (PMCAs). PMCAs extrude Ca^2+^ to the extracellular space, and knock‐out of Bsg considerably affects Ca^2+^ handling by PMCAs (Schmidt et al., 2017). Sphingolipid deficient synapses in the *lace* mutant have deficits in Bsg function which may in turn have an effect on Ca^2+^ dynamics via PMCA function.

Ablation of sphingolipid synthesis at a *Drosophila* model synapse supports a role for sphingolipids in maintenance of synaptic activity and regulation of synaptic structure. Our analysis also points to sphingolipid dependent regulation of synaptic structure via function of the small Ig‐domain protein Bsg. The precise regulation of synapse structure and function is a potent mechanism underlying synaptic plasticity and we suggest that the presence of sphingolipids at synapse may partially reflect this function.

## CONFLICT OF INTEREST

The authors declare no competing interests.

## AUTHOR CONTRIBUTIONS

Data were collected by R.J.H.W., R.R.R., M.P.F, and L.B. All Authors contributed toward the design, implementation and analysis of the experiments. Statistical analysis and assembly of figures was performed by R.J.H.W and R.R.R. The manuscript was written by R.J.H.W., R.R.R and S.T.S.

## Supporting information

Additional Supporting Information may be found online in the supporting information tab for this article.

Supporting Information Figure S1

Supporting Information Figure S2

Supporting Information Figure S3

Supporting Information Tables
