# Supplementary material for: Sphingolipids regulate neuromuscular synapse structure and function in Drosophila
Source: J Comp Neurol. 2018 Aug 2;526(13):1995–2009. doi: 10.1002/cne.24466 (PMC6175220; doi:10.1002/cne.24466)
Supplement: Supplementary file 4 — Supporting Information Tables [file CNE-526-1995-s004.docx]

| **Antibody** |  | **Immunogen** |  | **Manufacturer, Catalogue number, Species, RIDD** |  | **Concentration** | |
| --- | --- | --- | --- | --- | --- | --- | --- |
| **Anti-HRP (Cy3 Conjugated)** |  | Horseradish Peroxidase |  | Jackson ImmunoResearch Labs Cat# 123-165-021 RRID:AB_2338959, Goat, Polyclonal |  | 1:200 |  |
| **Anti-Synaptotagmin** |  | *Drosophila* Recombinant GST-dSyt1 |  | Sweeney Lab, (West et al., 2015), Rabbit, Polyclonal, RRID:AB_2713991 |  | 1:2000 |  |
| **Anti-NC82/Bruchpilot** |  | *Drosophila* C-terminal aa 1227-1740 |  | DSHB Cat# nc82 RRID:AB_2314867  Mouse, Monoclonal |  | 1:50 |  |
| **Anti-DLG** |  | *Drosophila* aa 439-756 |  | DSHB Cat# 4F3 anti-discs large RRID:AB_528203, Mouse, Monoclonal |  | 1:50 |  |
| **Anti-GluRIIB** |  | 15 C-terminal residues (-ASSAKKKKKTRRIEK) of predicted dGluRIIB |  | Marrus et al., (2004), Rabbit, Polyclonal |  | 1:2500 |  |
| **Anti-Basigin** |  | N-terminal synthetic peptide (QSLDKLVPNYD) |  | Besse et al., (2007), Rat, Polyclonal |  | 1:200 |  |
| **Anti-Syntaxin** |  | *Drosophila* Syntaxin |  | DSHB Cat# 8c3 RRID:AB_528484, Mouse, Monoclonal |  | 1:50 |  |
| **Anti-Na+/K+ ATPase** |  | Chicken ATPase, (Na(+) K(+) alpha subunit |  | DSHB Cat# a5 RRID:AB_2166869,  Mouse, Monoclonal |  | 1:100,000 |  |
| **HRP Conjugated anti-Mouse** |  |  |  | Cell Signaling Technology Cat# 7076 RRID:AB_330924, horse, polyclonal |  | 1:1000 |  |
| **HRP Conjugated anti-Rat** |  |  |  | Cell Signaling Technology Cat# 7077 RRID:AB_10694715, goat, polyclonal |  | 1:1000 |  |
|  |  |  |  |  |  |  |  |

**Supplementary**

**Supplementary Table 1. Antibodies**

**Supplementary Table 2.**

| ***Muscle 6*** | **EM (mV)** | **Rin  (MΩ)** | **mEJP  (mV)** | **mEJP f  (s^-1^)** | **Corrected EJP  (mV)** | **M1  (quanta)** | **Facilitation Index  (m5/m-1)** |
| --- | --- | --- | --- | --- | --- | --- | --- |
|  |  |  |  |  |  |  |  |
| **Wildtype** | -53.4 | 4.56 | 0.46 | 1.45 | 14.8 | 34.6 | 0.31 |
| N=8 larvae  n=12 muscles | (-57.6, -49.2) | (3.42, 5.70) | (0.37, 0.55) | (0.96, 1.95) | (12.2, 17.5) | (26.0, 43.2) | (0.19, 0.43) |
| **lace^5^/ lace^5^** | -55.4 | 4.06 | 0.65* | 2.05 | 24.5** | 39.3* | 0.00* |
| N=8 larvae  n=19 muscles | (-57.4, -53.4) | (3.15, 4.98) | (0.55, 0.76) | (1.62, 2.47) | (21.7, 27.2) | (33.7, 44.9) | (-0.04, 0.05) |
| **lace^5^/ lace^5^,UAS-lace;Tubulin-Gal4** | -53.3 | 5.27 | 0.7 | 2.4 | 14.2 | 21.3 | 0.19 |
| N=8 larvae  n= 21 muscles | (-58.4, -48.2) | (4.12, 6.42) | (0.59, 0.80) | (1.71, 3.08) | (12.0, 16.3) | (17.1, 25.6) | (0.03, 0.35) |

ANOVA with post-hoc Tukey comparison between groups

* P<0.05 compared with either Wildtype or Rescue

** P<0.05 compared with both Wildtype and Rescue

**Supplementary Table 3.**

| ***Muscle 7*** | **EM (mV)** | **Rin  (MΩ)** | **mEJP  (mV)** | **mEJP f  (s^-1^)** | **Corrected EJP  (mV)** | **M1  (quanta)** | **Facilitation Index  (m5/m-1)** |
| --- | --- | --- | --- | --- | --- | --- | --- |
|  |  |  |  |  |  |  |  |
| **Wildtype** | -51.8 | 5.983 | 0.62 | 1.06 | 14.8 | 20.9 | 0.43 |
| N=8 larvae  n=15 muscles | (-56.7, -46.9) | (4.90, 7.07) | (0.49, 0.74) | (0.79, 1.33) | (12.2, 17.5) | (14.6, 27.2) | (0.23, 0.64) |
| **lace^5^/ lace^5^** | -52.8 | 4.38 | 0.75 | 1.54* | 23.8** | 32.9** | 0.04* |
| N=8 larvae  n=16 muscles | (-55.3, -50.4) | (3.26, 5.51) | (0.67, 0.84) | (1.26, 1.83) | (19.1, 28.6) | (24.7, 41.0) | (-0.02, 0.11) |
| **lace^5^/ lace^5^,UAS-lace;Tubulin-Gal4** | -48.3 | 5.94 | 0.81 | 1.63 | 12.6 | 17.1 | 0.22 |
| N= 8 larvae  n=20 muscles | (-53.2, -43.4) | (4.62, 7.25) | (0.68, 0.93) | (1.33, 1.94) | (11.0, 14.7) | (13.2, 21.0) | (0.05, 0.38) |

ANOVA with post-hoc Tukey comparison between groups * P<0.05 compared with either Wildtype or Rescue

** P<0.05 compared with both Wildtype and Rescue
